# Efficacy of High-Dose Dexamethasone in Reducing the Symptoms of Postembolization Syndrome Following Prostatic Artery Embolization: Results of a Double-Blind Randomized Controlled Trial

**DOI:** 10.1007/s00270-023-03650-4

**Published:** 2024-01-17

**Authors:** Petra Svarc, Hein Vincent Stroomberg, Mikkel Taudorf, Klaus Brasso, Lars Lonn, Andreas Røder

**Affiliations:** 1 Department of Radiology, Rigshospitalet, Faculty of Health and Medical Sciences, University of Copenhagen, Blegdamsvej 9, 2100 Copenhagen, Denmark; 2 Department of Clinical Medicine, Faculty of Health and Medical Sciences, University of Copenhagen, Blegdamsvej 3B, 2100 Copenhagen, Denmark; 3 Copenhagen Prostate Cancer Center, Blegdamsvej 9, 2100 RigshospitaletCopenhagen, Denmark; 4 Section of Biostatistics, Department of Public Health, University of Copenhagen, Copenhagen, Denmark

**Keywords:** Dexamethasone, Prostate, Embolization, Therapeutic

## Abstract

**Purpose:**

To evaluate the efficacy of a single perioperative dose of dexamethasone in reducing postembolization syndrome following prostatic artery embolization.

**Materials and Methods:**

We conducted a single-center double-blind randomized controlled trial from March 2021 to May 2022 (NCT04588857). Participants were randomized to receive either i.v. 24 mg dexamethasone or saline. The primary outcome measures were temperature, pain, and quality of life in the first 5 days following prostatic artery embolization. Sample size of 60 patients was needed for the assessment of primary outcomes. Participants were followed for 6 months and assessed for a variety of secondary outcome measures including inflammatory markers and lower urinary tract symptoms severity.

**Results:**

Due to lack of clinical effect and mild symptoms in the control group, the trial was terminated early. 31 participants (16 dexamethasone vs. 15 control) were enrolled and analyzed. A difference in mean temperature was observed on day 1 (37.23 ± 0.64 °C control vs 36.74 ± 0.41 °C dexamethasone, *p* = 0.02, 95% CI 0.09–0.89). Difference in pain (score out of 10) was seen only on day 5 (1.48 ± 1.2 control vs. 2.9 ± 2.24 dexamethasone, *p* = 0.04, 95% CI − 2.78–− 0.04). A difference in C-reactive protein values was observed on day 2 (108 [54–161] mg/l control vs 10 [5–33] mg/l dexamethasone, *p* < 0.01). No significant differences in other outcomes were observed. No side effects were recorded.

**Conclusions:**

Twenty-four milligrams of dexamethasone bolus is safe but does not reduce postembolization syndrome following prostatic artery embolization.

**Graphical Abstract:**

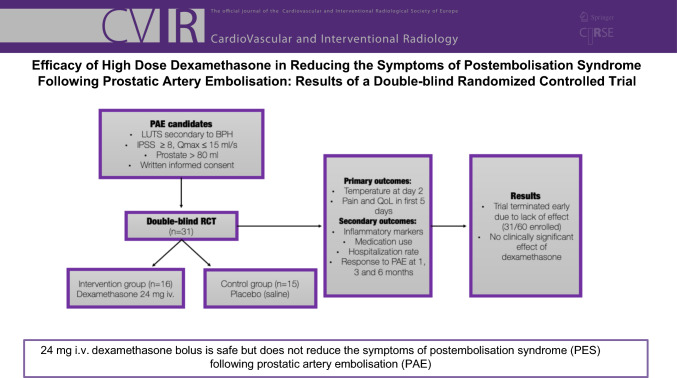

**Supplementary Information:**

The online version contains supplementary material available at 10.1007/s00270-023-03650-4.

## Introduction

Prostatic artery embolization (PAE) is a minimally invasive alternative for treatment of benign prostatic hyperplasia (BPH) and associated lower urinary tract symptoms (LUTS), with outcomes comparable to transurethral resection of the prostate [1, 2].

A common side effect of vascular embolization of solid organs, including prostate, is a collection of inflammation- and ischemia-related symptoms known as the postembolization syndrome (PES) [3, 4]. The symptoms most associated with PES are fever, pain, raised inflammatory parameters, nausea and vomiting, and in the case of PAE, dysuria and transient worsening of LUTS [4]. There is no consensus in the current literature on the definition of PES, and its reported incidence following PAE varies widely, from 0 to 100% [5]. Observations from our own group suggest that PES occurs in most patients with varying degrees of severity, ranging from only mild discomfort two to three days following PAE to hospital admission [6].

PES is ultimately a self-limiting condition that is managed with symptomatic therapy. However, PES can mimic septicemia with high fever, shivers, dysuria, and urgency. Leukocytosis and raised C-reactive protein (CRP) are also often observed in PES and can lead to overtreatment with intravenous antibiotics and hospital admission [3]. To avoid this, corticosteroids have been shown to reduce incidence and severity of PES following embolization of renal angiomyolipoma and uterine fibroids, as well as liver chemoembolization [7–9]. To date, no studies have been conducted in PAE.

In this study, we evaluated the efficacy of a single perioperative i.v.24 mg dose of dexamethasone (DEXA) in reducing PES following PAE in a double-blinded randomized clinical trial.

## Materials and Methods

### Study Design

This was a single-institution, double-blind, randomized controlled trial (RCT) comparing 24 mg intravenous (i.v.) dose of DEXA with placebo (isotonic solution of sodium chloride). The choice of 24 mg DEXA was based on the results of trials and meta-analyses exploring the anti-inflammatory effects of steroids given perioperatively at various doses [10–12]. The trial was conducted at a university-affiliated tertiary hospital with approximately 120 PAEs performed annually since January 2016. Participants were included from March 2021 to May 2022. Relevant approvals were granted by the regional Ethics Committee (H-20025910) and the Danish Medicines Agency (2020-000915-53). The trial was pre-registered on ClinicalTrials.gov (NCT04588857). The study design, intervention details, sample size calculation, and outcome measures have previously been reported in detail [13].

### Participants

Patients with BPH, a prostate > 80 ml measured by transrectal ultrasound, and LUTS, followed at the Department of Urology, Rigshospitalet, Copenhagen University Hospital, were invited to participate. Study flow and overview over inclusion and exclusion criteria can be found in the published protocol [13]. Inclusion criteria mimicked the standard PAE eligibility criteria at our institution. Exclusion criteria consisted of current urological contraindications to PAE, contraindications for catheter-based interventions and contraindications for high-dose steroid administration. Written informed consent was obtained from all participants prior to inclusion into the study.

### PAE Technique and Intervention

PAE was performed bilaterally following the standard “proximal embolization first, then embolize distal (PErFecTED)” technique [14]. Embolization was achieved using 300–500 μm Embosphere microspheres (Merit Medical). Participants were randomized with a 1:1 allocation ratio and a block size of 6 to receive either 24 mg DEXA (6 ml of 4 mg/ml solution) or 6 ml saline. Randomization was performed on the day of the intervention by a staff member not otherwise involved in the study or patient treatment. A radiographer blinded to the syringe content administered the medication through a peripheral vein immediately following the closure of the femoral access site. The participants were then transferred to the ward, where they were observed for 1–4 h, following standard procedure. All patients were prescribed prophylactic trimethoprim 200 mg twice daily for 3 days.

### Outcome Measures

Outcome measures collected during the study period can be seen in the published trial protocol [13]. All outcome measures during the first month following PAE, apart from blood tests, were self-reported by the participants in a diary provided on the day of the intervention. Participants were scheduled for a follow-up visit at three and 6 months following PAE, where outcome measures were collected by research staff.

Rectal temperature (in degrees Celsius) at 2 days following PAE was chosen as the first primary outcome measure, based on the results of a pilot study conducted prior to trial commencement, as described previously [13]. Morning rectal temperatures for both groups were compared, as it was hypothesized that they would be least influenced by medication intake and daily activity.

Additionally, mean postprocedural pain and QoL in the first 5 days post-PAE, measured as Pain Severity and Pain Interference scores on Brief Pain Inventory—Short Form (BPI-SF), were chosen as the second primary outcome measure [15].

A sample size of 28 patients per group was calculated to be necessary to detect a statistically significant reduction of 0.4 degrees Celsius in temperature at 2 days following PAE with a significance level of 0.05 and a power of 80%. To allow for dropouts, 30 patients per group were planned. Interim analysis was planned after 50% inclusion with a blinded data monitoring committee.

The effect of DEXA compared with placebo was assessed for the following secondary outcomes: postprocedural inflammatory response markers (CRP and prostate specific antigen (PSA)), medication usage (analgesics, antipyretics, antiemetics), incidence of nausea and vomiting, LUTS severity measured on International Prostate Symptom Score (IPSS), erectile function measured on International Index of Erectile Function—5 (IIEF-5), incidence of hospital admission, incidence of urinary tract infection (UTI), dysuria and acute urinary retention, mean (Qmean) and maximum urinary (Qmax) flow, residual urine and prostate volume (PV).

### Statistical Analysis

Results are expressed as mean ± standard deviation (SD) for continuous variables and number (percentage) for categorical variables. The independent t test was used for comparison of continuous outcomes when the assumptions of normality and homogeneity of variance were met, and the Mann–Whitney test was used when the assumptions were not met. Categorical variables were compared using *χ*^2^ test or Fisher exact test if numbers of expected values were < 5. Analysis of covariance (ANCOVA) was used to control for baseline values when analyzing follow-up measurements. A *P* value of < 0.05 (two-tailed) was considered statistically significant, and for the primary outcomes the effect sizes are stated with 95% confidence intervals (CIs). All statistical analysis was performed in R (version 3.6.2, The R Foundation for Statistical Computing) and figures were produced using GraphPad Prism version 9.4.1. for iOS (GraphPad Software, San Diego, California USA).

## Results

At interim analysis, no statistically significant difference in trial outcomes was demonstrated and the data monitoring committee recommended termination of the study and unblinding of the participants. We report data on 50% of planned number of participants that were included in the study.

### Patient Demographics

A total of 31 individuals were included in the study—15 in control group, and 16 in DEXA group. There were no dropouts. The two groups were well balanced in baseline patient demographics, with the exception of Qmean values, as seen in Table 1.

**Table 1:** Patient demographics at baseline

Variable^a^	Control (*n* = 15)	DEXA (*n* = 16)	*p* value
Age (y)	70 [66–75]	69 [65–75]	0.43
BMI (kg/m^2^)	27.4 ± 5.7	27.4 ± 4.9	0.99
PV (ml)	136 ± 65	126 ± 54	0.65
IPSS	21.0 ± 7.5	25.0 ± 7.2	0.11
Qmean (ml/s)	4.6 ± 1.6	3.0 ± 1.2	*0.01*
Qmax (ml/s)	8.8 ± 2.2	7.2 ± 3.1	0.09
Temperature (°C)	36.5 ± 0.3	36.4 ± 0.4	0.83

*BMI* body mass index, *IPSS* International Prostate Symptom Score, *PV* prostate volume; *Qmean* mean urinary flow, *Qmax* maximum urinary flow

^a^Age is presented as median [interquartile range]. Remaining variables are presented as mean ± standard deviation

### Primary Outcomes

**Fig. 1:**
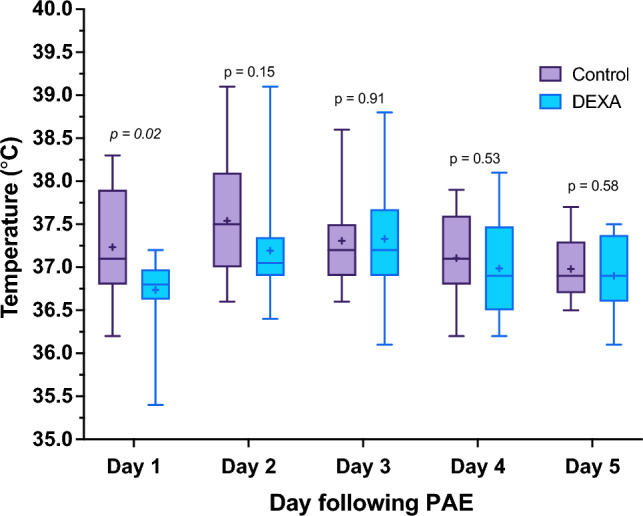
Daily morning temperature (°C) comparison for the two groups. Box = 25th and 75th percentiles; bars = minimum and maximum values (1.5* IQR); bold line = median; “ + ” = mean. Mann–Whitney test was used to compare the means of the two groups on Day 2, while independent *t* test was used for the remaining days. *DEXA* dexamethasone, *PAE* prostatic artery embolization

A difference in temperature was observed on day 1 following PAE, with mean temperature of 37.23 ± 0.64 °C in control group and 36.74 ± 0.41 °C in DEXA group (*p* = 0.02). No other statistically significant differences in temperature were observed, as seen in Fig. 1 and Supplementary Table 1.

**Fig. 2:**
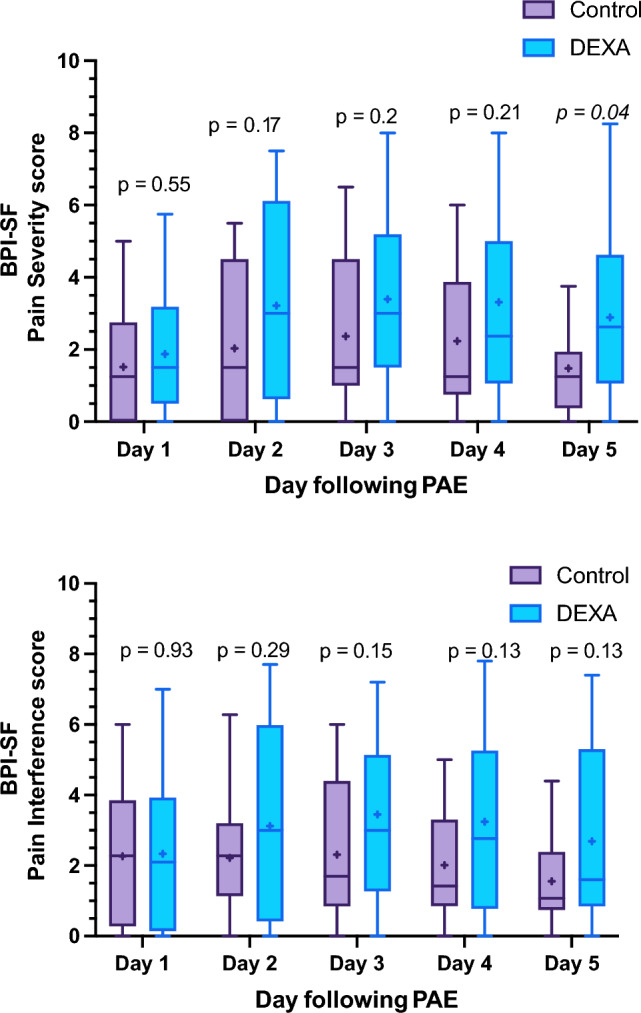
Daily BPI-SF score comparison for the two groups. Box = 25th and 75th percentiles; bars = minimum and maximum values (1.5* IQR); bold line = median: “ + ” = mean. Mann–Whitney test was used to compare the scores. Both scores are measured on a scale out of 10, where higher score indicated more pain (Pain Severity score) and lower quality of life (Pain Interference score). *BPI-SF* Brief Pain Inventory – Short Form, *DEXA* dexamethasone, *PAE* prostatic artery embolization

Less pain in control group, measured as Pain Severity score on BPI-SF (maximum score of 10, higher score indicates more severe pain), was seen on day 5 following PAE, with a score of 1.5 ± 1.2 in control vs 2.9 ± 2.2 in DEXA group (*p* = 0.04). No statistically significant differences in QoL, measured as Pain Interference score on BPI-SF (maximum score of 10, higher score indicates lower QoL), were seen. Daily overview over Pain Severity and Pain Interference scores can be seen in Fig. 2, as well as Supplementary Table 2.

### Secondary Outcomes

Secondary outcomes are presented in Table 2. All participants took some analgesics or antipyretics following PAE, but no statistically significant differences in dose were observed. We observed lower CRP values at day 2 in the DEXA group compared to control (108 [54–161] mg/l control vs 10 [5–33] mg/l, *p* < 0.01), supporting the anti-inflammatory effect of DEXA. A single patient in DEXA group was admitted to the hospital with severe PES, and one patient from the same group received intravenous antibiotic treatment for an UTI. Almost all patients reported dysuria (87% control vs 88% DEXA group), and two patients in the control group experienced acute urinary retention. No statistically significant difference was observed in reported nausea and vomiting. IPSS and IIEF-5 scores at all measurement timepoint were not statistically different. At 6 months following PAE, prostate volume decreased by approximately 50% in both groups compared to baseline. There was a statistically significant difference in baseline Qmean (*p* = 0.01) between the two groups, but there was no difference at follow-up when corrected for baseline values. No statistically significant difference in maximum urinary flow was observed at any timepoint.

**Table 2:** Comparison of secondary outcomes between the two groups

Outcome	Control (*n* = 15)	DEXA (*n* = 16)	*p* value
Intake of any medication during the 5 days	15 (100)	16 (100)	> 0.99
Daily paracetamol dose (mg)	1600 [1000–2900]	1750 [1175–2450]	0.93
Daily ibuprofen dose (mg)	0 [0–306]	80 [0–160]	0.27
CRP (mg/l)*			
Baseline	0 [0–4]	0 [0–4]	> 0.99
Day 2	108 [54–161]	10 [5–33]	< *0.01*
PSA (μg/l)*			
Baseline	11 [6.7–14]	6.7 [4.8–9.8]	0.50
Day 2	129 [103–162]	134 [112–150]	0.25
1 month	6.4 [5.2–10]	6.1 [5.3–10]	0.77
3 months	5 [3.9–7.3]	5.3 [3.2–7.5]	0.70
6 months	5.4 [4.5–7.9]	5.1 [3.9–8.8]	0.77
Hospital admission related to PES	0 (0)	1 (6)	0.33
UTI	0 (0)	1 (6)	0.33
Dysuria	13 (87)	14 (88)	0.94
Acute urinary retention	2 (13)	0 (0)	0.13
Nausea	11 (73)	9 (56)	0.32
Vomiting	4 (27)	2 (13)	0.31
IPSS score*			
Baseline	21.0 ± 7.5	25.0 ± 7.2	0.11
Day 2	21.1 ± 6.8	24.9 ± 6.9	0.34
Day 5	23.3 ± 7.2	23.9 ± 8.5	0.65
1 month	13.9 ± 4.9	13.3 ± 9.3	0.37
3 months	10.9 ± 7.4	11.1 ± 9.2	0.62
6 months	12.3 ± 6.2	14.2 ± 8.1	0.47
PV (cm^3^) *			
Baseline	136 ± 65	126 ± 54	0.65
3 months	89 ± 33	80 ± 27	0.37
6 months	72 ± 18	76 ± 26	0.21
IIEF-5*			
Baseline	15.7 ± 6.6	16.5 ± 6.3	0.75
1 month	16.4 ± 6.0	15.4 ± 6.3	0.40
3 months	16.6 ± 6.9	16.5 ± 6.4	0.80
6 months	15.7 ± 6.9	15.4 ± 7.8	0.49
Qmean (ml/s)*			
Baseline	4.6 ± 1.6	3.0 ± 1.2	*0.01*
3 months	4.7 ± 2.1	4.8 ± 2.9	0.90
6 months	4.7 ± 2.6	4.1 ± 1.7	0.45
Qmax (ml/s) *			
Baseline	8.8 ± 2.2	7.2 ± 3.1	0.09
3 months	11.9 ± 6.0	12.0 ± 5.9	0.95
6months	11.6 ± 6.6	11.3 ± 5.8	0.89
Residual urine (ml)*			
Baseline	60 [38–108]	92 [31–258]	0.31
3months	20 [0–57]	28 [5–57]	0.53
6 months	55 [50–80]	49 [12–95]	0.82

Categorical variables are expressed as *n* (%) and continuous variables as mean ± standard deviation or median [interquartile range]. *P values of variables with baseline measurements are corrected for baseline values

*CRP* C-reactive protein, *DEXA* dexamethasone, *IIEF*-5 International Index of Erectile Function – 5, *IPSS* International Prostate Symptom Score, *PES* postembolization syndrome, *PSA* prostate specific antigen, *PV* prostate volume, *Qmax* maximum urinary flow, *Qmean* mean urinary flow, *UTI* urinary tract infection

Statstically significant *p* values

## Discussion

We prospectively evaluated the efficacy of a single dose of 24 mg DEXA in reducing the incidence and severity of PES following PAE in a single-center, double-blind RCT. The trial was prematurely terminated after an interim analysis claimed futility of the outcome difference between the two groups. No clinically significant differences in either the primary or secondary outcomes were demonstrated, apart from CRP values at 2 days following PAE, which were lower in the DEXA group. Additionally, the proportion of participants in both groups who were febrile was lower than predicted by both the pilot study and previous clinical experience at our center.

No published guidelines for managing PES exist. Patients experiencing PES symptoms are generally treated symptomatically, with some centers empirically using corticosteroids at varying doses during or following PAE. While the anti-inflammatory and immunomodulatory effects of corticosteroids in reducing postoperative pain after traditional surgery are well studied, their efficacy in reducing PES symptoms was previously explored only in a limited number of studies in other anatomical sites. In uterine artery embolization (UAE) for symptomatic fibroids, a randomized controlled trial showed reduced need for fentanyl-based patient controlled analgesia and reduced incidence of nausea and vomiting in the group receiving supplementary dexmedetomidine, but the trial did not report if there was a significant difference in other symptoms of PES, such as fever [7]. Furthermore, a nonrandomized prospective study in 10 patients showed the effect of preoperative methylprednisolone in reducing fever and pain following renal angiomyolipoma embolization [8]. Additionally, methylprednisolone reduced the inflammatory response and pain after other interventional radiology procedures, such as endovascular aortic repair and transarterial chemoembolization of the liver (TACE) [9, 16]. On the other hand, two other TACE studies in 171 and 125 patients showed no effect of dexamethasone on the severity of PES symptoms [17, 18].

The heterogeneity of outcome definitions and different corticosteroid regimens represent major limitations of previous studies in terms of translating their results into clinical practice. This trial was designed to incorporate the broadest definition of PES into both the primary and secondary outcomes. As PAE is an outpatient procedure, single administration of a corticosteroid seemed to best mimic a real-life clinical setting. DEXA was chosen as it has the longest biological half-life of all corticosteroids, to account for the single dose given, as stated in the published protocol [13]. The relatively high dose of 24 mg was chosen for the same reason. In this trial, DEXA was given at the end of the procedure. It could be argued that DEXA could have had a more pronounced effect on outcome measures if it had been administered prior to commencing the procedure, as it would have hindered the inflammatory process from starting. The anti-inflammatory effect of DEXA was clearly visible from the difference in CRP values between the two groups 2 days after the procedure. The data failed to demonstrate that translated into a clinically relevant reduction in either PES symptoms or need for postprocedural medication. However, we observed a potential increase in pain and a lower quality of life in men assigned to the DEXA group. The influence of the placebo effect on the reported symptoms must also be considered and could have resulted in control group participants reporting less symptoms than usual [19].

During this study, a more detailed description of possible PES symptoms was given to the participants, and they were contacted by research staff more often than is standard practice. We found that this approach resulted in less patients contacting our emergency out-patient clinic compared to patients not enrolled in the study. This could indicate that a more holistic approach with good patient information and more frequent follow-ups is a significant factor in how patients experience symptoms of PES. More studies on this approach are warranted, as it might be more beneficial than a purely pharmaceutical viewpoint.

Limitations of the trial include that the trial was underpowered for the assessment of primary endpoints, due to early termination, and a larger sample size might demonstrate significant difference between groups. Yet, considering the mean temperature in the control group, any significant differences are unlikely to be clinically relevant considering tradeoffs related to active treatment. Additionally, due to clinical study design and the nature of the procedure, primary endpoints were self-measured by the participants, which might have resulted in an increase in measurement errors. To minimize potential measurement errors the morning temperature was taken for analysis. Moreover, the measurement error would be similar in both arms of the trial and are therefore unlikely to influence the conclusion of the trial. Further, all procedures were conducted using the relatively large particles, 300–500 microns, and there is some evidence suggesting that the use of smaller particles could cause more pronounced and more frequent PES symptoms [20, 21]. Finally, no direct measurements of the extent of prostatic tissue infarction were collected during this trial. This is not a part of the standard PAE protocol at our institution, and this trial was designed to mimic the clinical reality as close as possible. It could be argued that patients with more extensive areas of infarction could experience more severe PES and would thus benefit from treatment with corticosteroids. However, it is questionable if administering corticosteroids routinely to all patients to potentially minimize PES in those with extensive infarctions would be considered good clinical practice.

## Conclusion

This study could not demonstrate a clinically significant difference in the severity of PES symptoms following PAE in participants receiving a single 24 mg dose of DEXA compared to placebo. Due to missing effect and clinical relevancy, the trial was terminated prematurely and is thus underpowered. We do not believe a larger trial would demonstrate a temperature difference that would justify active treatment with steroids.

### Supplementary Information

Below is the link to the electronic supplementary material.

Supplementary file 1 (DOCX 14 kb)

Supplementary file 2 (DOCX 16 kb)
