# Supplementary material for: Efficacy of High-Dose Dexamethasone in Reducing the Symptoms of Postembolization Syndrome Following Prostatic Artery Embolization: Results of a Double-Blind Randomized Controlled Trial
Source: Cardiovasc Intervent Radiol. 2024 Jan 17;47(5):632–9. doi: 10.1007/s00270-023-03650-4 (PMC11074011; doi:10.1007/s00270-023-03650-4)
Supplement: Supplementary file 1 — (DOCX 14 kb) [file 270_2023_3650_MOESM1_ESM.docx]

**Supplementary table 1** – Morning temperature comparison for the two groups

| Time | Morning temperature (°C) | | Mean  difference | *p* value | 95% CI |
| --- | --- | --- | --- | --- | --- |
|  | Control  (n=15) | DEXA  (n=16) |  |  |  |
| Baseline | 36.47 ± 0.36 | 36.44 ± 0.42 | 0.03 | 0.83 | -0.25 – 0.31 |
| Day 1 | 37.23 ± 0.64 | 36.74 ± 0.41 | 0.49 | *0.02* | 0.09 – 0.89 |
| Day 2 | 37.54 ± 0.70 | 37.19 ± 0.59 | 0.35 | 0.15 | -0.13 – 0.83 |
| Day 3 | 37.31 ± 0.56 | 37.33 ± 0.70 | 0.02 | 0.91 | -0.49 – 0.44 |
| Day 4 | 37.11 ± 0.49 | 36.99 ± 0.6 | 0.12 | 0.53 | -0.26 – 0.50 |
| Day 5 | 36.98 ± 0.35 | 36.90 ± 0.45 | 0.08 | 0.58 | -0.22 – 0.38 |
| Average of 5 days | 37.23 ± 0.47 | 37.00 ± 0.40 | 0.23 | 0.21 | -0.12 – 0.52 |

Temperature expressed as mean ± standard deviation. 95% CI = 95% confidence interval.

DEXA = dexamethasone.
