# Supplementary material for: Efficacy of High-Dose Dexamethasone in Reducing the Symptoms of Postembolization Syndrome Following Prostatic Artery Embolization: Results of a Double-Blind Randomized Controlled Trial
Source: Cardiovasc Intervent Radiol. 2024 Jan 17;47(5):632–9. doi: 10.1007/s00270-023-03650-4 (PMC11074011; doi:10.1007/s00270-023-03650-4)
Supplement: Supplementary file 2 — (DOCX 16 kb) [file 270_2023_3650_MOESM2_ESM.docx]

**Supplementary table 2** – Comparison of Pain Severity scores and Pain Interference scores on BPI-SF for the two groups

| Time | Pain Severity score ^a^ | | Mean  difference | *p* value | 95% CI |
| --- | --- | --- | --- | --- | --- |
|  | Control  (n=15) | DEXA  (n=16) |  |  |  |
| Day 1 | 1.52 ± 1.57 | 1.88 ± 1.77 | 0.36 | 0.55 | -1.57 – 1.73 |
| Day 2 | 2.10 ± 1.90 | 3.22 ± 2.70 | 1.12 | 0.17 | -2.90 – 0.54 |
| Day 3 | 2.40 ± 2.00 | 3.40 ± 2.30 | 1.00 | 0.20 | -2.65 – 0.60 |
| Day 4 | 2.23 ± 1.92 | 3.31 ± 2.60 | 1.08 | 0.21 | -2.80 – 0.65 |
| Day 5 | 1.48 ± 1.23 | 2.90 ± 2.24 | 1.42 | *0.04* | -2.78 – -0.04 |
| Average of 5 days | 1.88 ± 1.36 | 2.94 ± 2.10 | 1.10 | 0.10 | -2.35 – 0.23 |

| Time | Pain Interference score ^b^ | | Mean  difference | *p* value | 95% CI |
| --- | --- | --- | --- | --- | --- |
|  | Control  (n=15) | DEXA  (n=16) |  |  |  |
| Day 1 | 2.27 ± 1.93 | 2.34 ± 2.16 | 0.07 | 0.93 | -1.57 – 1.43 |
| Day 2 | 2.22 ± 1.65 | 3.12 ± 2.80 | 0.90 | 0.29 | -2.60 – 0.81 |
| Day 3 | 2.31 ± 2.00 | 3.45 ± 2.30 | 1.14 | 0.15 | -2.73 – 0.45 |
| Day 4 | 2.00 ± 2.61 | 3.25 ± 1.62 | 1.25 | 0.13 | -2.85 – 0.38 |
| Day 5 | 1.55 ± 1.31 | 2.70 ± 2.50 | 1.15 | 0.13 | -2.63 – 0.35 |
| Average of 5 days | 2.04 ± 1.36 | 2.97 ± 2.20 | 0.39 | 0.18 | -2.30 – 0.46 |

Scores expressed as mean ± standard deviation.

95% CI = 95% confidence interval; BPI-SF = Brief Pain Inventory – Short Form; DEXA = dexamethasone.

^a^ Score expressed on a scale out of 10, higher score indicates more severe pain.

^b^ Score expressed on a scale out of 10, higher score indicates lower daily quality of life.
